# Antioxidant and Antimicrobial Activities of the Extracts from Different *Garcinia* Species

**DOI:** 10.1155/2021/5542938

**Published:** 2021-06-18

**Authors:** Nguyen Hoai Nguyen, Minh Tan Nguyen, Hiep Dinh Nguyen, Phuoc Dien Pham, Ut Dong Thach, Binh T. D. Trinh, Ly T. T. Nguyen, Son V. Dang, Anh Thu Do, Bich Hang Do

**Affiliations:** ^1^Faculty of Biotechnology, Ho Chi Minh City Open University, Ho Chi Minh City, Vietnam; ^2^NTT Hi-Tech Institute, Nguyen Tat Thanh University, Ho Chi Minh City, Vietnam; ^3^Faculty of Pharmacy, Ton Duc Thang University, Ho Chi Minh City, Vietnam; ^4^Faculty of Chemistry, University of Science VNU-HCM, 227 Nguyen Van Cu, Ho Chi Minh City, Vietnam; ^5^Vietnam National University Ho Chi Minh City, Ho Chi Minh City, Vietnam; ^6^Institute of Tropical Biology, VAST, 85 Tran Quoc Toan, Ho Chi Minh City, Vietnam; ^7^English Faculty, Foreign Trade University-Ho Chi Minh City Campus, Ho Chi Minh City, Vietnam

## Abstract

**Background:**

*Garcinia* is a large genus which has promising bioactivities. However, the properties of many *Garcinia* species have not been investigated thoroughly.

**Aim:**

To determine the antioxidant and antimicrobial capabilities of the extracts from different *Garcinia* species. *Methodology*. Six *Garcinia* species, including *Garcinia fusca*, *Garcinia hopii*, *Garcinia planchonii*, *Garcinia nigrolineata*, *Garcinia gaudichaudii*, *and Garcinia tinctoria* were extracted using *n*-hexane, ethyl acetate, and methanol, producing *n*-hexane extract (HE), ethyl acetate extract (EAE), and methanol extract (ME). After that, the total polyphenol content was evaluated using Folin–Ciocalteu assay. DPPH, hydroxyl radical scavenging, and total antioxidant capacity assays were performed to test the antioxidant activity. Subsequently, the antimicrobial activities against Gram-positive (*Staphylococcus aureus*, *Bacillus subtilis*) and Gram-negative (*Escherichia coli*, *Pseudomonas aeruginosa*) bacterial strains were assessed using Kirby Bauer and the broth microdilution methods.

**Results:**

Many *Garcinia* extracts contained high total polyphenol content consisting of ME of *G. hopii* ad *G. tinctoria*, and EAE of *G. planchonii* and *G. tinctoria*. The EAE of *G. tinctoria* showed effective antioxidant capacity (IC_50_ = 1.5 *µ*g/mL). Additionally, the EAE of *G. gaudichaudii* was effective against Gram-positive bacteria with minimal inhibition concentration (MIC) of 15.625–25 *µ*g/mL whereas ME of *G. planchonii* was effective against both Gram-positive bacteria (MIC = 160 *µ*g/mL) and Gram-negative bacteria (MIC = 75 *µ*g/mL).

**Conclusion:**

Several extracts of *Garcinia* species demonstrated valuable antioxidant and antimicrobial properties.

## 1. Introduction

The genus *Garcinia* belonging to the Clusiaceae family is a sizable group of plants. It consists of over 200 species mainly distributing in the world's tropics, chiefly in Asia, Africa, and Polynesia. They are evergreen polygamous trees, shrubs, and herbs. Many species of *Garcinia* have been proven highly nutritious and utilized as food, supplemental products, and nutritional supplements. The most widely studied is *G. mangostana* or mangosteen, the extract of which contains a high amount of isoprenylated xanthones exerting anticancer, analgesic, neuroprotective, antidiabetic, and hypolipidemic effects [1]. Another species of *Garcinia* is *G. cambogia*, whose extract containing high hydroxycitric acid which is used as a weight-loss supplement by reducing fat accumulation [2, 3]. Moreover, the supplement of *G. cambogia* also helps to reduce inflammation and glucose intolerance [4]. Other species of *Garcinia* have also been investigated; those include *G. celebica*, *G. nigrolineata*, *G. cowa*, *G. indica*, *G. schomburgkiana* Pierre., *G. preussii*, and *G. fusca* [1, 5–8]. Specifically, the extract *G. celebica* leaves exerted anticancer activities by enhancing apoptosis on MCF-7 human breast cancer cell lines [5]. In addition, the leaf extract of *G. celebica* and extracted compound, catechin, exhibited antiproliferation on *Plasmodium falciparum* by oxidative stress induction [9]. Likewise, xanthones collected from the root of *G. fusca* inhibited *α*-glucosidase, making it a candidate for antidiabetes [10] whereas quinone and xanthones isolated from the extracts of *G. nigrolineata* show high antibacterial activity [6, 11, 12]. Another outstanding feature is that the fruit rind of *G. indica* possesses both antioxidant and hepatoprotective properties [13].

In modern societies, the increasing level of stress, air pollution, food toxicity, and so forth causes reactive oxygen species production, leading to DNA damage, which gives rise to several chronic and degenerative diseases such as antiaging, anti-inflammatory, antiatherosclerosis, and anticancer [14, 15]. Antioxidants are substances that inhibit the initiation or propagation of oxidative chain reactions, scavenge free radicals, quench singlet oxygen, and reducing agents, resulting in the reduction of oxidative stress for the cell [16, 17]. Therefore, finding substances and extracts with high antioxidant capacity is a crucial issue.

The plan is believed to be a crucial source of natural antioxiadants and antimicrobial activity. Firstly, these natural antioxidants, especially polyphenols and carotenoids, are capable of scavenging free radicals or stabilizing free reactive oxygen species to prevent oxidative stress within the cell environment. Studies demonstrated that several *Garcinia* species possessed the high antioxidant ability, including *G. mangostana L.*, *G. indica*, *G. cambogia*, *G. atroviridis* [13, 18–21]. The antioxidant activity of these extracts has been applied to treat various related diseases. Secondly, with regard to antimicrobial activity, the plant extracts might be derived from phenolics, terpenoids, essential oil, alkaloids, lectins, polypeptides, and polyacetylenes [22–24]. Several *Garcinia* has demonstrated the capability of exhibiting antimicrobial activity. For instance, the extracts of *G. cambogia* leaves and *G. indica* fruits showed interesting anthelmintic and antibacterial activities against earthworm and Gram-positive bacteria, respectively [25, 26].

In Vietnam, there are several *Garcinia* species grown wildly, of which their bioactivities have not been clearly elucidated. In this study, six *Garcinia* species including *G. fusca*, *G. hopii*, *G. planchonii*, *G. nigrolineata*, *G. gaudichaudii*, and *G*. *tinctoria* were collected and extracted using different solvents. Subsequently, the polyphenolic content, the antioxidation, and the antimicrobial capability of the extracts were examined.

## 2. Materials and Methods

### 2.1. Materials

The plant samples were collected from different Vietnam provinces and identified by Dr. Son V. Dang, the curator of the VNM Herbarium, Institute of Tropical Biology, Ho Chi Minh City, Vietnam. Voucher specimens were deposited in the Natural Product and Medicinal Chemistry Lab, University of Science, VNU-HCM. Data of sample collection are shown in Table 1.

Solvents such as *n*-hexane, ethyl acetate, methanol, and dimethyl sulfoxide (DMSO) were purchased from Sigma-Aldrich (St Louis, MO, USA). Folin–Ciocalteu and 2, 2-diphenyl-1-picrylhydrazyl (DPPH) were obtained from Sigma-Aldrich (St Louis, MO, USA). Mueller-Hinton Broth and Mueller-Hinton Agar were acquired from Thermo Fisher Scientific (Massachusetts, USA).

### 2.2. Methods

#### 2.2.1. The Preparation of *Garcinia* Extracts

One hundred grams of each plant material was air-dried, grounded, and then subjected to Soxhlet extraction with 350 mL of methanol to yield a concentrated solution. Methanol was then removed under reduced pressure using a rotary evaporator system to obtain an initial extract. The extract was then subjected to solid-phase extraction, where the sample was thoroughly washed with 5 L of *n*-hexane, 10 L of ethyl acetate, and 10 L of methanol, respectively. After concentration, *n*-hexane extract (HE), ethyl acetate extract (EAE), and methanol extract (ME) were attained. Data of extract preparation are described in Table 1.

#### 2.2.2. The Measurement of Total Phenolic Content

Total phenolic content (TPC) of *Garcinia* extracts was determined using Folin–Ciocalteu assay, following the protocol of Singleton et al. [27]. In particular, 800 *µ*L of HE, EAE, and ME at the concentration of 50 *μ*g/mL were mixed with 100 *μ*L of Folin–Ciocalteu reagent (diluted 4 times), followed by an addition of 100 *μ*L of 20% (w/v) sodium carbonate. After being mixed carefully, the mixture was kept in the dark at room temperature for 30 mins. The absorbance of these samples was measured at 760 nm using a spectrophotometer (Thermo Scientific, Massachusetts, USA). Gallic acid was used as a reference. Gallic acid at different concentrations (200, 100, 50, 25, 12.5, 6.25, 3.125, and 1.56 *μ*g/mL) was applied following the aforementioned protocol to prepare a standard curve. TPC was expressed as milligrams of gallic acid equivalent (GAE) per gram of the dry extract.

#### 2.2.3. DPPH Assay

The free radical scavenging activity of the extracts was evaluated using DPPH assay according to the method described by Shen et al. [28]. The extracts were diluted in 0.1 mM DMSO to various concentrations from 0 to 200 *μ*g/mL. 0.5 mL of the extracted was added to 1.5 mL of 0.1 mM DPPH which was dissolved completely in methanol. The mixtures were shaken vigorously and incubated in the dark at room temperature for 30 mins. Then, the absorbance was measured at 517 nm using a spectrophotometer. Ascorbic acid was used as a positive control. The capability of scavenging the DPPH radical was calculated using the following formula:

(1) $$\begin{array}{c} \%\text{ inhibition }\left(\text{\%}I\right)=\left\{\left(\frac{A_{0}-A_{1}}{A_{0}}\right)*100\right\}. \end{array}$$

*A*_0_ is the absorbance of the control reaction and *A*_1_ is the absorbance in the presence of the extracts or positive control.

#### 2.2.4. Determination of Hydroxyl Radical Scavenging Assay

The hydroxyl radical scavenging activity of the extracts was assessed according to the protocol of Klein et al. [29]. 90 *μ*L of the extracts with the concentrations ranging from 0 to 1,000 *μ*g/mL was supplemented with 45 *μ*L of 8 mM FeSO_4_.7H_2_O, 63 *μ*L of 5.7 mM salicylic acid, and 72 *μ*L of 6 mM H_2_O_2_. The mixture was incubated at 37°C for 30 mins before being applied onto a spectrophotometer to measure the absorbance at 562 nm. Ascorbic acid was used as a positive control. The percentage of the hydroxyl scavenging activity was calculated using the following formula:

(2) $$\begin{array}{c} \%\text{ OH}-\text{scavenging activity }=\;\left[1-\left(\frac{A_{\mathrm{sample}}-A_{\text{sample blank}}}{A_{\mathrm{control}}-A_{\text{control blank}}}\right)\right]\times 100. \end{array}$$

*A*_sample blank_ or *A*_control blank_ is the absorbance value of sample or control without salicylic acid.

*A*_sample_ or *A*_control_ is the absorbance value of the sample or control with salicylic acid.

#### 2.2.5. Determination of Total Antioxidant

The total antioxidant capacity (TAC) of the extracts was estimated following the method reported by Wan C. et al. [30]. 0.1 mL of the extracts at the concentration of 200 *μ*g/mL was added with 0.9 mL of 0.6 mol/L sulfuric acid, 28 mmol/L sodium phosphate, and 4 mmol/L ammonium molybdate. The mixture was incubated at 90°C for 90 mins using a water bath. After cooling down to room temperature, the samples were centrifuged at 5000 rpm for 5 mins to obtain the supernatants. The absorbance of the supernatants was measured at 695 nm using a spectrophotometer. Ascorbic acid at a 0–125 *μ*g/mL concentration was used to prepare a standard curve. The activity of extracts was expressed as ascorbic acid equivalent (AAE) mg per g of the extracts.

#### 2.2.6. Kirby Bauer Assay

The antibacterial activity of the extracts was tested using Kirby Bauer diffusion method [31]. Briefly, the extract at a concentration of 100 mg/mL was prepared in DMSO. Different bacteria including Gram-positive (*Staphylococcus aureus, Bacillus subtilis*) and Gram-negative (*E. coli, Pseudomonas aeruginosa*) were cultured in Mueller–Hinton broth for 3 h at 37°C, 200 rpm shaking. The cell density was determined by measuring optical density at 600 nm using spectrophotometer. The bacterial suspensions were diluted to 10^8^ CFU/mL, then spread on the surface of Mueller-Hinton agar plates using sterile swabs. 6 mm Ø wells were created and 20 *µ*L the extracts were then supplemented into each well. The plates were incubated at 4°C for 2 h to allow the diffusion of the active compounds in the medium. The plates were incubated at 37°C for 16–18 h. Plates treated with 20 *µ*L DMSO were used as negative controls and ciprofloxacin (5 *µ*g/well) was used as positive controls. The antibacterial activity was determined by measuring the diameter of the inhibition zone to the nearest millimeter.

#### 2.2.7. Determination of Minimum Inhibitory Concentration

The minimum inhibitory concentration (MIC) of the extracts was carried out by using the broth microdilution method [31]. The test was performed using sterile polystyrene 96-well plates. 100 *µ*L of autoclaved Mueller-Hinton broth supplemented with different concentrations of the extracts was added to each well. Subsequently, 5 *µ*L of the bacteria including *Staphylococcus aureus*, *Bacillus subtilis*, *Pseudomonas aeruginosa*, and *Escherichia coli* at the concentration of approximately 10^8^ CFU/mL was added. The plate was shaken and incubated at 37°C for 24 h. 0.1% DMSO was used as a negative control and ciprofloxacin was used as a positive control. MIC was defined as the lowest concentration of the extract at which the microorganisms showed no visible growth.

#### 2.2.8. Statistical Analysis

All the data are presented as the mean ± standard error (SE). Student's *t*-test was used to determine the statistical significance of group means. All tests were two-sided and *p* values less than 0.05 were considered statistically significant.

## 3. Results

### 3.1. Determination of Total Phenolic Content

TPC of different extracts was estimated using Folin–Ciocalteu method. Gallic acid was used as a standard. The results were expressed as microgram of GAE per milligram of the extracts (Table 2). In consequence, many *Garcinia* extracts showed high TPC. Notably, the ME of *G. hopii* and *G. tinctoria* and EAE of *G. planchonii* and *G. tinctoria* showed high TPC with the values of 60.1, 70.8, 61, and 74.1 *µ*g GAE/mg, respectively. Among tested *Garcinia* species, *G. tinctoria* exhibited high TPC in both EAE and ME. Conversely, there was low TPC in all extracts of *G. fusca*, *G. gaudichaudii*, and *G. nigrolineata*.

### 3.2. DPPH Assay

**Figure 1:**
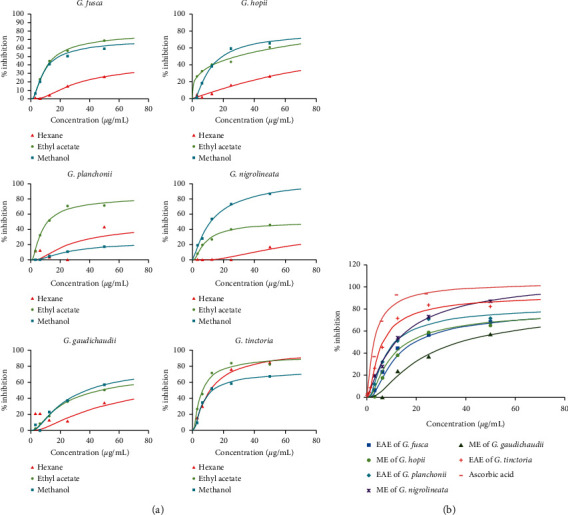
The free radical scavenging capability of extracts of *Garcinia* species using DPPH assay. (a) The activities of various extracts. (b) The activities of different species. The extracts were supplemented with DPPH (dissolved in methanol), then shaken vigorously, and incubated in dark at room temperature for 30 mins. Subsequently, the absorbance was measured at 517 nm using a spectrophotometer. Ascorbic acid was used as a positive control and 0.1 mM DMSO was used as a negative control. The most bioactive extract of each species was chosen to compare with others.

The free radical scavenging of the extracts was estimated using DPPH assay. The results were expressed in percentage values of inhibition of radicals (Figure 1). Both EAE of G. *tinctoria* and ME of *G. nigrolineata* comparably scavenged the free radicals with the inhibition of approximately 83.5%–87% (IC_50_ = 1.5 *µ*g/mL and 2.5 *µ*g/mL, resp.). The EAE of *G. planchonii* and *G. fusca* and ME of *G. hopii* showed comparable scavenging property, approximately 71.45%, 68.6%, and 65%, respectively. No obvious inhibition was observed in other extracts. Ascorbic acid was used as a positive control, exhibiting the scavenging of 95% with the IC_50_ of 0.78 *µ*g/mL.

### 3.3. Hydroxyl Radical Scavenging Assay

**Figure 2:**
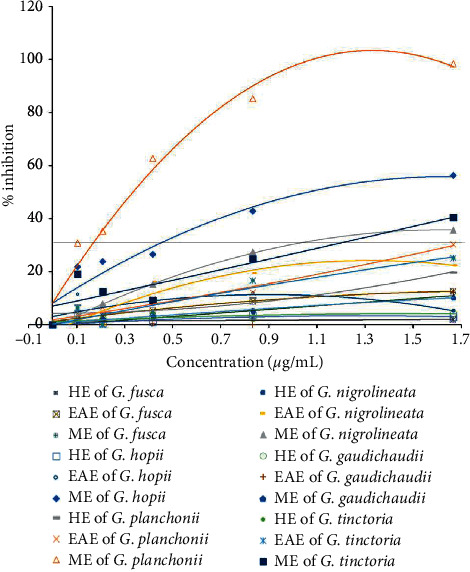
The hydroxyl radical scavenging capability of extracts of different *Garcinia* species. The extracts were supplemented with FeSO_4_.7H_2_O, salicylic acid, and H_2_O_2,_ followed by being mixed and incubated at 37°C for 30 mins before applied onto a spectrophotometer to measure the absorbance at 562 nm. Ascorbic acid was used as a positive control and 0.1 mM DMSO was used as a negative control.

The scavenging of OH^−^ was estimated following the procedure as described by Smirnoff and Cumbes [32]. The present study revealed the high OH^−^ radical scavenging activity of ME of *G. planchonii* (98%) with the IC_50_ of 178 *µ*g/mL, followed by ME of *G. hopii* (56%). Low inhibition was attained in other extracts. Ascorbic acid was used as a positive control which showed the maximum scavenging of 94% with the IC_50_ of 5.5 *µ*g/mL (Figure 2).

### 3.4. Total Antioxidant Capacity Assay

The TAC of the extracts was evaluated using phosphomolybdate assay, measuring the ability of a sample to destroy a free radical by transferring an electron to the latter. Ascorbic acid was used to provide a standard curve and the TAC was expressed as milligram of AAE per gram of the extract.  Table 3 indicates the high TAC of *G. tinctoria*. Particularly, the TAC of ME, EAE, and HE of *G. tinctoria* was 265.9, 169.8, and 135.6 mg AAE/g extract, respectively. Comparably, the TACs of ME of *G. hopii* and EAE of *G. planchonii* were 208.2 and 160.1 mg AAE/g extract, respectively.

### 3.5. The Antimicrobial Activity

The antimicrobial property of the extracts was evaluated using diffusion Kirby Bauer assay. The diameters of the inhibition zone were described in Table 4. Subsequently, the antimicrobial extracts were screened for MIC using the microdilution method.  Table 5 showed that EAE of *G. gaudichaudii* was effective against Gram-positive bacteria (*S. aureus* and *B. subtilis*) with an MIC of approximately 15.625–25 *µ*g/mL whereas ME of *G. planchonii* was effective against both Gram-positive bacteria (*S. aureus* and *B. subtilis*) (MIC = 160 *µ*g/mL) and Gram-negative bacteria (*E. coli*, *P. aeruginosa*) (MIC = 75 *µ*g/mL). However, the MICs of both extracts were higher compared to that of ciprofloxacin (MIC = 0.25 *µ*g/mL and 0.016 *µ*g/mL against Gram-positive and Gram-negative bacteria, subsequently).

## 4. Discussion

In this study, we collected six species of *Garcinia* including *G. fusca*, *G. planchonii*, *G. hopii*, *G. tinctoria*, *G. nigrolineata*, and *G. gaudichaudii* and then extracted them using different solvents, named hexane, ethyl acetate, and methanol. The phytochemicals of the extract relating to polyphenol were measured and the bioactivity including antioxidant and antimicrobial capacity was determined. The results indicated that ME and EAE of *G. tinctoria* showed promising antioxidant property while EAE of *G. gaudichaudii* and ME of *G. planchonii* possessed high antimicrobial activity.

Phenolic compounds are secondary metabolites containing at least one phenol unit in the structure. They are phytochemicals found in many plants which have a function as substances protecting plants from oxidative stress by neutralizing the free radicals by donating hydrogen, reducing and chelating metal, and capturing singlet oxygen [33]. Additionally, these metabolites break the free radical chain reaction by forming a relatively stable phenoxy-radical intermediate [34]. They are antioxidants which are used to treat various diseases relating to oxidative stress including cancer, cardiovascular diseases, Alzheimer's disease, and aging-related diseases [35, 36]. Therefore, isolating the extracts containing high phenolic compounds is required. In the present study, of the six, *G. tinctoria* showed the highest TPC (74.1 *µ*g GAE/mg) (Table 2). Compared to HE, ME and EAE possessed higher TPC. The difference in solvent polarity determines the components in the extracts. The high polarity (methanol) and medium polarity (ethyl acetate) solvents enabled the isolation of more effective polyphenol compounds. Previous studies revealed the capacity of ethyl acetate and methanol in the extraction of high bioactive compounds [37–39]. Therefore, the choice of a suitable solvent is important in the isolation of natural extracts or compounds.

The DPPH assay is a frequently used technique to determine the radical scavenging capacity of plant-based extracts because of its quick and responsive features, which involve simple conventional laboratory equipment. The results showed that ME and EAE exhibited higher free radical scavenging ability compared to HE. Among them, EAE of *G. tinctoria* possessed the strongest free radical scavenging capacity with the inhibition of approximately 83.5% at IC_50_ of 1.5 *µ*g/mL (Figure 2). The TPC results were in accordance with the antioxidant activity determined by DPPH and TAC assays (Table 2, Figure 1) which demonstrated that the antioxidant activity of *G. tinctoria* was mainly due to hampering free radicals of phenolic compounds in the extract. Many studies mentioned effective free radical inhibition of the phenolic-rich extraction [40–42]. However, the polyphenol content was not in accordance with the hydroxyl scavenging capacity (Table 2 and Figure 2). In particular, ME of *G. planchonii* showed the strongest hydroxyl scavenging activity with low polyphenol content (approximately 17.16 *µ*g GAE/mg). Therefore, other compounds might contribute to this activity of the extract.

In terms of antimicrobial activity, ME of *G. planchonii* arrested the growth of both Gram-positive and Gram-negative bacterial strains while EAE of *G. gaudichaudii* hampered the proliferation of Gram-positive bacteria (Tables 4 and 5). However, the activities of both extracts were lower compared to those of ciprofloxacin (positive control). The poorer activity may be due to the interference with the active compounds of other components in the extract. Mostly the extracts or compounds target Gram-positive strains better than Gram-negative strains. The difference might be explained by the distinction in the structure of peptidoglycan, presence of outer membrane, receptors or lipids, cross-linking, and the activity of autolytic enzymes that determine the penetration, binding, and action of the compounds [43]. A study by Sunitha Janardhanan et al. demonstrated that crude chloroform extract of *G. mangostana* pericarp effectively inhibited Gram-positive bacteria including *Streptococcus mutans*, *Streptococcus sanguis*, *Streptococcus salivarius*, *Streptococcus oralis*, and *Lactobacillus acidophilus* [44]. Besides, other natural extracts or compounds also exhibited a higher sensitivity to Gram-positive bacteria [45–47].

## 5. Conclusions

This study was conducted by extracting different *Garcinia* species grown in Vietnam using various solvents. The total polyphenol content and bioactivity including antioxidant and antimicrobial activities were evaluated. Our results demonstrated that the EAE of *G. tinctoria* showed promising antioxidant capacity. In the antimicrobial assays, ME of *G. planchonii* was efficient against both Gram-positive bacteria (*S. aureus* and *B. subtilis*) (MIC = 160 *µ*g/mL) and Gram-negative bacteria (*E. coli*, *P. aeruginosa*) (MIC = 75 *µ*g/mL) while EAE of *G. gaudichaudii* was effective against Gram-positive bacteria (*S. aureus* and *B. subtilis*) with the MIC of approximately 15.625–25 *µ*g/mL. Taken together, our results elucidated the antioxidation and antimicrobial property of several *Garcinia* extracts.

## Figures and Tables

**Table 1:** Summarization of sample collection and extract preparation of various species of genus *Garcinia.*

*Garcinia* species	Collecting part	Collecting region	Collecting date	Weight of extracts (g)	
*G. fusca*	Roots	Dong Nai	March 2015	Hexane	2.90
				Ethyl acetate	6.67
				Methanol	4.97

*G. hopii*	Stems	Khanh Hoa	April 2015	Hexane	0.64
	Leaves			Ethyl acetate	0.97
				Methanol	0.82

*G. planchonii*	Barks	Khanh Hoa	April 2015	Hexane	10.19
				Ethyl acetate	9.00
				Methanol	5.61

*G. nigrolineata*	Barks	Dong Nai	March 2016	Hexane	7.33
				Ethyl acetate	1.32
				Methanol	3.37

*G. gaudichaudii*	Barks	Phu Quoc	May 2016	Hexane	4.86
				Ethyl acetate	0.78
				Methanol	2.21

*G. tinctoria*	Barks	Dong Nai	May 2016	Hexane	9.26
				Ethyl acetate	3.61
				Methanol	3.35

**Table 2:** The total polyphenolic content of various species of genus *Garcinia*.

*Garcinia* species	Extracts	Total polyphenolic content (*µ*g QE/mg extract)
*G. fusca*	HE	20.58 ± 2.3
	EAE	45.18 ± 4.4
	ME	35.17 ± 2.5

*G. hopii*	HE	22.14 ± 5.1
	EAE	20.22 ± 2.4
	ME	60.09 ± 4.3

*G. planchonii*	HE	39.53 ± 2.5
	EAE	60.99 ± 5.6
	ME	17.16 ± 2.1

*G. nigrolineata*	HE	17.89 ± 2.5
	EAE	34.78 ± 3.4
	ME	29.99 ± 2.4

*G. gaudichaudii*	HE	12.34 ± 1.5
	EAE	41.59 ± 4.3
	ME	15.76 ± 3.4

*G. tinctoria*	HE	27.79 ± 3.8
	EAE	74.12 ± 4.9
	ME	70.84 ± 3.6

**Table 3:** The total antioxidant capacity of the extracts of different *Garcinia* species.

*Garcinia* species	Extracts	Total antioxidant capacity (mg AAE/g extract)
*G. fusca*	HE	0
	EAE	130.16 ± 5.3
	ME	34.56 ± 2.1

*G. hopii*	HE	2.57 ± 0.5
	EAE	91.77 ± 2.4
	ME	209.22 ± 7.5

*G. planchonii*	HE	0
	EAE	160.16 ± 5.5
	ME	35.08 ± 6.3

*G. nigrolineata*	HE	5.43 ± 1.2
	EAE	111.13 ± 4.5
	ME	87.26 ± 8.2

*G. gaudichaudii*	HE	4.35 ± 1.2
	EAE	70.48 ± 5.4
	ME	76.29 ± 7.3

*G. tinctoria*	HE	135.65 ± 8.4
	EAE	169.84 ± 4.5
	ME	265.97 ± 9.4

**Table 4:** The inhibition zone diameter of extracts of different *Garcinia* species on bacterial strains.

*Garcinia* species	Extracts	Inhibition zone diameter (mm)			
		*S. aureus*	*B. subtilis*	*P. aeruginosa*	*E. coli*
Ciprofloxacin		17	15	23	32
*G. fusca*	HE	8	—	8	—
	EAE	12.5 ± 2.5	11 ± 2.3	8.5 ± 1.5	—
	ME	—	—	—	—

*G. hopii*	HE	—	—	—	—
	EAE	9 ± 1.2	—	9 ± 1.3	—
	ME	10.5 ± 2.3	—	7 ± 1	—

*G. planchonii*	HE	8 ± 1.4	—	8 ± 1.5	—
	EAE	12 ± 1.7	—	—	—
	ME	17 ± 2.5	16 ± 1.2	15 ± 1.5	20 ± 1.2

*G. nigrolineata*	HE	—	—	—	—
	EAE	9 ± 1.3	—	9 ± 1.8	8 ± 1.4
	ME	—	—	9 ± 1.4	—

*G. gaudichaudii*	HE	—	—	—	—
	EAE	17 ± 1	14 ± 0.7	7.5 ± 1.5	—
	ME	8 ± 1.7	—	8 ± 2.3	—

*G. tinctoria*	HE	10 ± 2.1	—	—	—
	EAE	12 ± 1.9	—	—	—
	ME	10 ± 0.9	—	—	—

**Table 5:** The MICs of extracts of different *Garcinia* species on bacterial strains.

The extracts	MICs on bacterial strains (*µ*g/mL)			
	*S. aureus*	*B. subtilis*	*E. coli*	*P. aeruginosa*
Ciprofloxacin	0.25	0.25	0.016	0.016
EAE of *G. fusca*	120	50	100	100
ME of *G. hopii*	140	200	200	100
ME of *G. planchonii*	160	160	75	75
EAE of *G. gaudichaudii*	15.625	25	100	100

## Data Availability

The datasets used and/or analyzed during the current study are available from the corresponding author on reasonable request.

## Abbreviations

DPPH: 2, 2-Diphenyl-1-picrylhydrazyl
IC_50_: Half-maximal inhibitory concentration
MIC: Minimal inhibition concentration
VNU-HCM: Vietnam National University-Ho Chi Minh City
DMSO: Dimethyl sulfoxide
HE: *n*-Hexane extract
EAE: Ethyl acetate extract
ME: Methanol extract
GAE: Gallic acid equivalent
TAC: Total antioxidant capacity
AAE: Ascorbic acid equivalent
SE: Standard error
TPC: The polyphenolic content.
